# Applying deep learning-based ensemble model to [^18^F]-FDG-PET-radiomic features for differentiating benign from malignant parotid gland diseases

**DOI:** 10.1007/s11604-024-01649-6

**Published:** 2024-09-10

**Authors:** Masatoyo Nakajo, Daisuke Hirahara, Megumi Jinguji, Mitsuho Hirahara, Atsushi Tani, Hiromi Nagano, Koji Takumi, Kiyohisa Kamimura, Fumiko Kanzaki, Masaru Yamashita, Takashi Yoshiura

**Affiliations:** 1 Department of Radiology, Graduate School of Medical and Dental Sciences, Kagoshima University, 8-35-1 Sakuragaoka, Kagoshima, 890-8544 Japan; 2 Department of Management Planning Division, Harada Academy, 2-54-4 Higashitaniyama, Kagoshima, 890-0113 Japan; 3 Department of Radiology, Nanpuh Hospital, 14-3 Nagata, Kagoshima, 892-8512 Japan; 4 Department of Otolaryngology Head and Neck Surgery, Graduate School of Medical and Dental Sciences, Kagoshima University, 8-35-1 Sakuragaoka, Kagoshima, 890-8544 Japan; 5 Department of Advanced Radiological Imaging, Graduate School of Medical and Dental Sciences, Kagoshima University, 8-35-1 Sakuragaoka, Kagoshima, 890-8544 Japan

**Keywords:** Parotid gland diseases, [^18^F]-FDG, Positron emission tomography computed tomography, Machine learning

## Abstract

**Objectives:**

To develop and identify machine learning (ML) models using pretreatment 2-deoxy-2-[^18^F]fluoro-D-glucose ([^18^F]-FDG)-positron emission tomography (PET)-based radiomic features to differentiate benign from malignant parotid gland diseases (PGDs).

**Materials and methods:**

This retrospective study included 62 patients with 63 PGDs who underwent pretreatment [^18^F]-FDG-PET/computed tomography (CT). The lesions were assigned to the training (*n* = 44) and testing (*n* = 19) cohorts. In total, 49 [^18^F]-FDG-PET-based radiomic features were utilized to differentiate benign from malignant PGDs using five different conventional ML algorithmic models (random forest, neural network, k-nearest neighbors, logistic regression, and support vector machine) and the deep learning (DL)-based ensemble ML model. In the training cohort, each conventional ML model was constructed using the five most important features selected by the recursive feature elimination method with the tenfold cross-validation and synthetic minority oversampling technique. The DL-based ensemble ML model was constructed using the five most important features of the bagging and multilayer stacking methods. The area under the receiver operating characteristic curves (AUCs) and accuracies were used to compare predictive performances.

**Results:**

In total, 24 benign and 39 malignant PGDs were identified. Metabolic tumor volume and four GLSZM features (GLSZM_ZSE, GLSZM_SZE, GLSZM_GLNU, and GLSZM_ZSNU) were the five most important radiomic features. All five features except GLSZM_SZE were significantly higher in malignant PGDs than in benign ones (each *p* < 0.05). The DL-based ensemble ML model had the best performing classifier in the training and testing cohorts (AUC = 1.000, accuracy = 1.000 vs AUC = 0.976, accuracy = 0.947).

**Conclusions:**

The DL-based ensemble ML model using [^18^F]-FDG-PET-based radiomic features can be useful for differentiating benign from malignant PGDs.

**Second abstract:**

The DL-based ensemble ML model using [^18^F]-FDG-PET-based radiomic features can overcome the previously reported limitation of [^18^F]-FDG-PET/CT scan for differentiating benign from malignant PGDs. The DL-based ensemble ML approach using [^18^F]-FDG-PET-based radiomic features can provide useful information for managing PGD.

**Supplementary Information:**

The online version contains supplementary material available at 10.1007/s11604-024-01649-6.

## Introduction

The majority of salivary gland tumors presented in the parotid gland accounting for approximately 60–70% of cases [1, 2] and the malignancy rate of parotid gland tumors was approximately 25% [1, 2]. Differentiating between benign and malignant parotid gland diseases (PGDs) before treatment assists in surgical preparation and patient consultation [3]. Fine-needle aspiration cytology (FNAC) and core needle biopsy (CNB) are both valuable tools in the differential diagnosis of PGDs [4]. Studies have indicated that FNAC has a sensitivity of around 80% and a specificity close to 95% for distinguishing between benign and malignant PGDs [5, 6]. Nonetheless, there are instances where FNAC results are non-diagnostic, with approximately 15% of cases falling into this category [6]. Alternatively, a recent meta-analysis found that CNB exhibits pooled sensitivity and specificity of 94% and 98%, respectively, for distinguishing benign from malignant PGDs [7]. Additionally, the non-diagnostic rate was reported to be 3.6% [7]. CNB is noted for its higher sensitivity compared to FNAC, with similar specificity and a lower rate of non-diagnostic cases [4]. Although generally safe, CNB carries some risks, such as tumor seeding and facial nerve damage, particularly with the use of thicker needles [8]. It also requires local anesthesia, making it more time-consuming than FNAC [4]. Consequently, a noninvasive diagnostic approach that can distinguish between benign and malignant PGDs would be helpful for pretreatment risk assessment and prediction in patients with PGDs.

Positron emission tomography (PET)/X-ray computed tomography (CT) scan, using the tracer 2-deoxy-2-[^18^F]fluoro-D-glucose ([^18^F]-FDG), is applied in oncology to assess metabolic glucose activity [9]. However, high [^18^F]-FDG uptake can also be observed in benign tumors such as Warthin’s tumor and earlier research has explored the limitations of [^18^F]-FDG-PET/CT scans for differentiating between benign and malignant tumors in the salivary glands [10].

Various mathematical methods in radiomics are used to extract numerous quantitative features, which help in obtaining useful biological information [11]. A few studies have examined the use of [^18^F]-FDG-PET-based radiomic features for predicting treatment response or prognosis in patients with salivary grand cancers [12, 13]. However, to the best of our knowledge, no study has examined the usefulness of [^18^F]-FDG-PET-based radiomic features in differentiating benign from malignant PGDs. Moreover, recent studies have reported the clinical applications of machine learning (ML) methods using [^18^F]-FDG-PET-based radiomics to overcome classification issues such as differential diagnosis or to predict treatment outcomes and prognosis in head and neck tumors [14–24].

Therefore, this study examined the usefulness of ML analyses, including the deep learning (DL)-based ensemble model with pretreatment [^18^F]-FDG-PET-based radiomic features, in differentiating benign from malignant PGDs.

## Materials and methods

### Patients

Our institutional review board approved this retrospective study, waiving the need for written informed consent. From January 2011 to December 2022, 72 consecutive patients with suspected or confirmed primary parotid gland tumors underwent a pretreatment [^18^F]-FDG-PET/CT scan. Clinical records were reviewed to identify eligible patients for analysis.

Inclusion criteria included: (1) patients with a pathological diagnosis from biopsy or surgical resection and (2) those with primary tumors showing visible uptake on the PET/CT scan. Exclusion criteria were: (1) patients with very small primary tumors (volume of interest [VOI] < 64 voxels) making accurate texture analysis difficult, and (2) those with incomplete clinical or follow-up data.

### Imaging protocols

A PET/CT scan was conducted using two different whole-body PET/CT scanners: Discovery 600-M PET/CT scan (GE Healthcare, Milwaukee, WI, USA) between January 2011 and 2018, and the Discovery MI (GE Healthcare) between 2018 and December 2022. Patients were required to fast for a minimum of 5 h prior to the examination, with an average plasma glucose level of 108 mg/dL (range: 81–178 mg/dL). The [^18^F]-FDG (FDG Scan; Nihon Medi-Physics, Tokyo, Japan) was administered intravenously, and the emission scan commenced 1 h post-injection of [^18^F]-FDG, with an average dose of 218 ± 35 MBq (range: 157–286 MBq), following the acquisition of CT scan data (slice thickness: 3.75 mm; pitch: 1.375 mm; 120 keV; and auto mA: 40–100 mA, adjusted according to body mass). The data acquisition time per bed position was 2.5 min (covering 7–11 positions) and data with attenuation correction were acquired. For the Discovery 600-M scanner, image reconstruction was achieved using a three-dimensional ordered subset expectation–maximization algorithm (image matrix size: 192 × 192; 16 subsets, two iterations; voxel size: 3.125 × 3.125 × 3.27 mm^3^; VUE Point Plus). For the Discovery MI scanner, a Bayesian penalized likelihood reconstruction algorithm (image matrix size: 192 × 192; voxel size: 2.60 × 2.60 × 2.78 mm^3^; penalization factor: 700; Q. Clear) was applied, incorporating the point spread function.

### Image and radiomic feature analyses

Two radiologists, each with 13 and 20 years of experience in [^18^F]-FDG-PET/CT scan interpretation respectively, reviewed the scans to ascertain if the primary lesion showed abnormal [^18^F]-FDG uptake, which was defined as exceeding the background activity in the surrounding tissues. They were aware of the study’s objectives but blinded to the clinical and pathological details. A third radiologist, with 18 years of experience in [^18^F]-FDG-PET/CT reading, conducted quantitative analyses of the visible primary lesions. This radiologist manually delineated the volume of interest (VOI) on an appropriate fused axial image, ensuring it encompassed the entire visible primary lesion while excluding adjacent physiological [^18^F]-FDG-avid structures. The boundaries of the VOI were defined using a 40% threshold of the maximum standardized uptake value (SUVmax) [25]. The LIFEx software (version 6.00) [26] was utilized to extract 49 radiomic features from the PET images (Supplemental Table 1). These features fell into five categories: shape and first-order features, gray-level co-occurrence matrix, neighborhood gray-level difference matrix, gray-level run-length matrix, and gray-level size-zone matrix (GLSZM). LIFEx calculates textural features only for VOIs consisting of at least 64 voxels. Using absolute resampling, both the VOI and SUV were divided into discrete bins to lessen the correlation between textural features and noise impact, as well as matrix sizes [27]. For resampling the PET component, 64 bins were used, with the SUVs interval set from 0 to 20, and voxel size resampled to 3.0 × 3.0 × 3.0 mm^3^. Consequently, a bin size of 0.3 SUV was utilized for analyzing the PET component. Voxels with an SUV greater than 20 were grouped into the highest bin [27].

**Table 1:** Characteristics of patients with parotid gland diseases (*n* = 63)

Characteristic	Training cohort	Testing cohort	*p* value^a^
Number	44	19	
Age (years) (mean, range)	64, 28–91	66, 29–90	0.31
Sex			0.85
Male	29	13	
Female	15	6	
Differentiation of benignity and malignancy			0.89
Benign parotid gland disease	17	7	
Malignant parotid gland disease	27	12	

Pathological diagnosis of parotid gland diseases	Training cohort	Testing cohort	Total number
Benign parotid gland disease			
Inflammatory disorder	7	2	9
Warthin’s tumor	5	3	8
Pleomorphic adenoma	1	2	3
Basal cell adenoma	2	0	2
Schwannoma	1	0	1
Myoepithelial tumor	1	0	1
Total number of benign parotid gland diseases	17	7	24
Malignant parotid gland disease			
Parotid gland carcinoma	20	8	28
Mucoepidermoid carcinoma	7	1	8
Carcinoma ex pleomorphic adenoma	6	2	8
Salivary duct carcinoma	3	0	3
Squamous cell carcinoma	2	1	3
Adenoid cystic carcinoma	1	1	2
Undifferentiated carcinoma	1	1	2
Epithelial-myoepithelial carcinoma	0	1	1
Secretory carcinoma	0	1	1
Malignant lymphoma	7	3	10
Capicua transcriptional repressor-rearranged sarcoma	0	1	1
Total number of malignant parotid gland diseases	27	12	39
Total number of parotid gland diseases	44	19	63

^a^Comparison of the training and testing cohorts

Due to the use of two distinct PET scanners, post-reconstruction harmonization of all PET parameters was carried out using the ComBat harmonization method implemented in R software (https://github.com/Jfortin1/ComBatHarmonization) [28], known for its effectiveness in PET imaging (details of ComBat harmonization method were described in the supplemental material) [29].

### Histological analyses

All clinical records, including pathological reports, were reviewed, and parotid gland tumors were classified according to the World Health Organization classification of head and neck tumors [30].

### ML approach

A total of 49 radiomic features were employed to distinguish between benign and malignant PGDs using ML techniques. The data were stratified by event and then randomly split into training (70%) and testing (30%) cohorts. To address data imbalance, the synthetic minority oversampling technique was applied to the training cohort [31]. Given the relatively small sample size (*n* = 63), it was crucial to reduce the feature set to avoid overfitting. In the training dataset, feature selection was performed using recursive feature elimination (RFE). This method systematically removes the least important features and refines the model iteratively until the desired number of features is retained [32]. RFE continuously evaluates and eliminates features to identify those contributing most effectively to model performance [32]. Following standard practice, the selected features constituted less than 10% of the total sample size [33]. Consequently, five features were identified for building the ML models. To further mitigate overfitting, a resampling technique known as k-fold cross-validation was implemented [34, 35]. Previous studies suggest that tenfold cross-validation is commonly used, especially when the dataset is neither too large nor too sparse [35]. In this study, a tenfold cross-validation approach was adopted to minimize overfitting in the training cohort. The study utilized commonly used ML algorithms for binary classification, specifically random forest (RF), neural network, k-nearest neighbors (kNN), logistic regression (LR), and support vector machine (SVM). Conventional ML analysis was conducted using Orange version 3.24.1 (Bioinformatics Laboratory, University of Ljubljana, Ljubljana, Slovenia), an open-source data mining and visualization tool [36].

The deployment of a DL-based ensemble model using AutoGluon, an AutoML framework, entails the integration of various DL architectures and the aggregation of each model’s predictions to improve accuracy [37–39]. AutoGluon, an open-source automated ML library developed by Amazon, serves as a tool for automating ML tasks, allowing for the automatic selection and training of ML models [40, 41]. AutoGluon leverages the training of numerous different models and their fusion, resulting in an extremely effective method. The analysis process consists of three main steps: (1) train individual models in the stacking phase, (2) implement bagging, and (3) perform multilayer stacking, which involves combining each model’s output with the data to perform stacking again, training multiple models, and using a linear model for the final output. In this study, the models within the AutoGluon framework include ExtraTrees, kNN, LightGBM, LinearModel, NeuralNetFastAI, NeuralNetTorch, RF, XGBoost, and WeightedEnsemble models that consolidate full-layer results. The specific parameters used were bag_folds: 5, stack_levels: 2, and presets: best_quality.

To evaluate the models’ predictive capabilities, receiver operating characteristic (ROC) curve analysis was used and the area under the ROC curve (AUC) was measured. The performance metrics calculated included AUC, accuracy, F1 score, precision (positive predictive value), and recall (sensitivity) averaged over all classes. The F1 score, also known as the F score or F measure, represents the harmonic mean of precision and recall [42]. Each ML algorithm generated a probability score (ranging from 0 to 1) for malignant PGDs. Each model’s performance in prediction was independently assessed in the test set through the metrics of AUC, accuracy, F1 score, precision, and recall. Additionally, diagnostic indices such as sensitivity, specificity, positive predictive value (PPV), and negative predictive value (NPV) were calculated for the test cohort.

### Statistical analysis

To evaluate differences between two quantitative variables or compare categorical data, the Mann–Whitney U test or the chi-square test was employed as appropriate. ROC analysis was utilized to assess the diagnostic performance of each parameter in predicting malignant PGDs. The DeLong method was applied to determine statistically significant differences between AUCs [43]. Diagnostic indices such as sensitivity, specificity, PPV, NPV, and accuracy were compared using either McNemar’s test or the chi-square test. Data were reported as median and interquartile range (IQR). All *p*-values were two-sided, with *p*-values < 0.05 considered statistically significant. Statistical analyses were conducted using MedCalc (MedCalc Software, Mariakerke, Belgium).

## Results

### Characteristics of the patients

Of the 72 patients, 7 who did not undergo surgery were excluded from the study. Of the remaining 65 patients, 3 (2 benign diseases; one inflammatory disorder and one pleomorphic adenoma, and one malignant lymphoma) were further excluded due to small tumor sizes (VOI < 64 voxels). Finally, 62 patients (41 men and 21 women; age: mean ± SD: 65 ± 17 [range: 28–91] years) with 63 lesions were eligible for analysis. Table 1 shows the clinical characteristics of the study populations. Thirty-six patients underwent the PET/CT examinations on the Discovery 600-M scanner, while the remaining 26 patients underwent the PET/CT examinations on the Discovery MI scanner, respectively. Of 63 lesions, 24 were benign PGDs (*n* = 9, inflammatory disorders; *n* = 8, Warthin’s tumors; *n* = 3 pleomorphic adenomas; *n* = 2, basal cell adenomas; *n* = 1, schwannoma; and *n* = 1, myoepithelial tumor). Further, 39 cases involved malignant PGDs (*n* = 28, parotid gland carcinomas [*n* = 8, mucoepidermoid carcinomas; *n* = 8, carcinoma ex pleomorphic adenomas; *n* = 3, salivary duct carcinomas; *n* = 3, squamous cell carcinomas; *n* = 2, adenoid cystic carcinomas; *n* = 2, undifferentiated carcinomas; *n* = 1, epithelial-myoepithelial carcinoma; and *n* = 1, secretory carcinoma], *n* = 10 malignant lymphomas, and *n* = 1, capicua transcriptional repressor-rearranged sarcoma).

### ML models for predicting malignant parotid tumors

Of the 44 parotid gland lesions in the training cohort, 17 were benign and 27 were malignant. Of the 19 parotid gland lesions in the testing cohort, 7 were benign and 12 were malignant. No significant difference was observed in the distribution of age, sex, differentiation of benignity and malignancy between the training and testing cohorts (each, *p* > 0.05) (Table 1).

Regarding the differentiation between benign and malignant PGDs, five radiomic features including metabolic tumor volume (MTV), GLSZM_zone size entropy (GLSZM_ZSE), GLSZM_small zone emphasis (GLSZM_SZE), GLSZM_gray level nonuniformity (GLSZM_GLNU), and GLSZM_zone size nonuniformity (GLSZM_ZSNU) were selected by the RFE feature selection method for constructing the ML model. All, except GLSZM_SZE, exhibited significant differences between benign and malignant diseases. Malignant diseases had a significantly higher MTV (median: 13.16 vs. 6.62,* p* = 0.024), GLSZM_ZSE (median: 5.50 vs. 4.78, *p* = 0.012), GLSZM_GLNU (median: 8.39 vs. 4.94, *p* = 0.020), and GLSZM_ZSNU (median: 49.42 vs. 25.12, *p* = 0.039) than benign diseases (Table 2). On the other hand, conventional SUV-related parameters including SUVmax and SUVmean exhibited no significant differences between benign and malignant PGDs (benign vs. malignant: SUVmax: median: 9.09 vs. 10.00,* p* = 0.21; SUVmean: median: 4.74 vs. 5.4, *p* = 0.14) (Table 2).

**Table 2:** Comparison of the selected radiomic features and SUV-related parameters for differentiation between benign and malignant parotid gland diseases

Feature	Patients with benign parotid gland diseases (*n* = 24)			Patients with malignant parotid gland diseases (*n* = 39)			*p* value
	Median	IQR	Range	Median	IQR	Range	
*Selected features by the RFE*							
MTV (mL)	6.62	3.26–17.94	2.12–39.44	13.16	5.56–32.02	1.87–84.94	0.024
GLSZM_ZSE	4.78	4.30–5.27	3.59–6.03	5.50	4.87–5.89	3.10–6.36	0.012
GLSZM_SZE	0.59	0.48–0.65	0.20–0.86	0.60	0.41–0.72	0.28–0.92	0.80
GLSZM_GLNU	4.94	3.36–11.63	1.67–27.36	8.39	4.67–17.66	2.33–71.79	0.020
GLSZM_ZSNU	25.12	9.82–41.24	1.53–302.69	49.42	14.33–144.70	2.27–747.91	0.039
*SUV-related parameters*							
SUVmax	9.09	5.99–10.69	3.39–29.69	10.00	6.70–15.92	2.69–43.61	0.21
SUVmean	4.74	2.51–6.88	1.89–16.21	5.40	3.62–10.45	1.68–30.16	0.14

*RFE *recursive feature elimination, *IQR* interquartile range, *MTV* metabolic tumor volume, *GLSZM* gray-level size-zone matrix, *ZSE* zone-size entropy, *SZE* small-zone emphasis, *GLNU* gray-level nonuniformity, *ZSNU *zone-size nonuniformity

Table 3 presents the diagnostic performance of each conventional ML model and the DL-based ensemble model in the training and testing cohorts for differentiating benign from malignant PGDs. Within the training cohort, all but the LR model among the five conventional ML models achieved AUC values above 0.80 for predicting malignant PGDs, with a range of 0.885–1.000. The DL-based ensemble model had a perfect classification performance with an AUC of 1.000.

**Table 3:** Diagnostic performance of each machine learning model and deep learning-based ensemble model using the top five radiomic features for differentiating benign from malignant parotid gland diseases in the training and testing cohorts

Model	Training cohort (average over classes)					Testing cohort								
						Average over classes				Predicting malignant parotid gland diseases				
	AUC	F1	Precision	Recall	Accuracy	AUC	F1	Precision	Recall	Sensitivity	Specificity	PPV	NPV	Accuracy
RF	0.996	0.963	0.966	0.963	0.963	0.726	0.644	0.680	0.684	91.6% (11/12)	28.6% (2/7)	68.8% (11/16)	66.7% (2/3)	68.4% (13/19)
Neural network	1.000	1.000	1.000	1.000	1.000	0.905	0.895	0.895	0.895	91.6% (11/12)	85.7% (6/7)	91.6% (11/12)	85.7% (6/7)	89.5% (17/19)
kNN	0.885	0.833	0.834	0.833	0.833	0.815	0.780	0.791	0.789	91.6% (11/12)	57.1% (4/7)	78.6% (11/14)	80.0% (4/5)	78.9% (15/19)
LR	0.656	0.720	0.722	0.730	0.722	0.726	0.732	0.731	0.737	83.3% (10/12)	57.1% (4/7)	76.9% (10/13)	66.7% (4/6)	73.7% (14/19)
SVM	0.870	0.700	0.822	0.778	0.778	0.929	0.793	0.814	0.789	75.0% (9/12)	85.7% (6/7)	90.0% (9/10)	66.7% (6/9)	78.9% (15/19)
Ensemble DL	1.000	1.000	1.000	1.000	1.000	0.976	0.960	0.923	1.000	100% (12/12)	85.7% (6/7)	92.3% (12/13)	100% (6/6)	94.7% (18/19)

*RF *random forest, *kNN* k-nearest neighbors, *LR* logistic regression, *SVM* support vector machine, *DL* deep learning, *AUC* area under the receiving operating characteristic curve

In the testing cohort, three of five conventional ML models (neural network, kNN, and SVM) achieved an AUC of > 0.80 (range: 0.815–0.929). Regarding the remaining two conventional ML models, the performance of RF was poorer in the testing cohort (AUC of 0.726) than in the training cohort (AUC of 0.996). The performance of LR did not achieve an AUC value of > 0.80 (AUC: 0.726). In contrast, the DL-based ensemble model had the highest AUC value (0.976). The AUC of the DL-based ensemble model was significantly higher than that of the RF (*p* = 0.030) and LR (*p* = 0.046) models (Supplemental Table 2). There were no significant differences in terms of AUC between the DL-based ensemble model and the other three conventional ML models (each *p* > 0.05). There were no significant differences in the diagnostic indices—sensitivity, specificity, PPV, NPV, and accuracy—between all conventional ML and DL-based ensemble models (each *p* > 0.05) (Supplemental Table 2). However, the DL-based ensemble model had the highest accuracy (94.7% [18/19]). Furthermore, the classification performance in the testing cohort was almost comparable to that observed in the training cohort (AUC: training cohort, 1.000; testing cohort, 0.976).

**Fig. 1:**
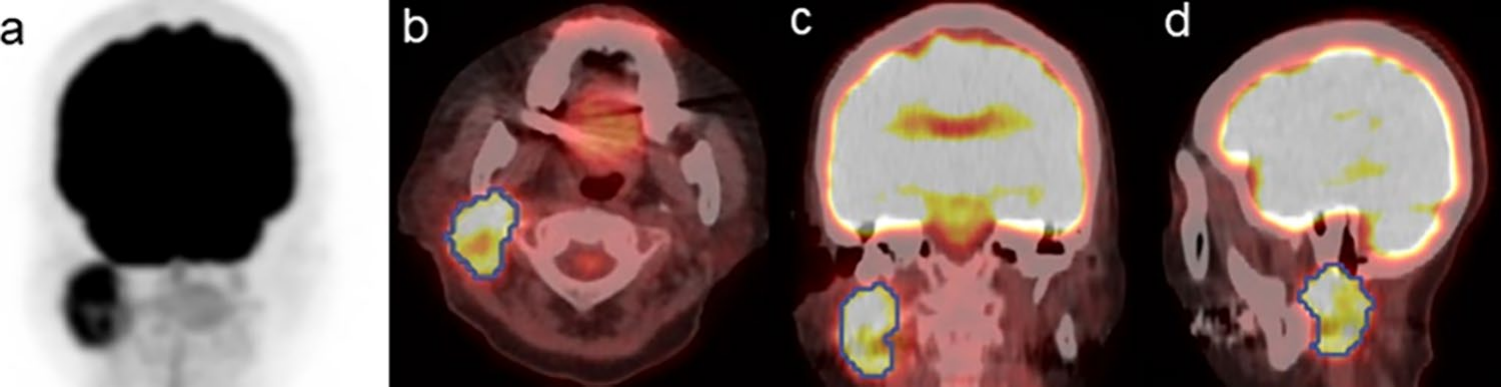
A 74-year-old female patient with a right malignant parotid gland tumor (squamous cell carcinoma). Pretreatment 2-deoxy-2-[^18^F]fluoro-d-glucose ([^18^F]-FDG) positron emission tomography/computed tomography scan images (maximum intensity projection [MIP] (**a**), transaxial (**b**), coronal (**c**), and sagittal (**d**)) revealed focal [^18^F]-FDG uptake in the primary lesion. The blue line represents the border of the volume of interest. The calculated probability score for predicting malignancy (positive ≥ 0.5) was 0.61 in the DL-based ensemble model. Thus, the DL-based ensemble model can predict the malignant parotid gland disease in this case

**Fig. 2:**
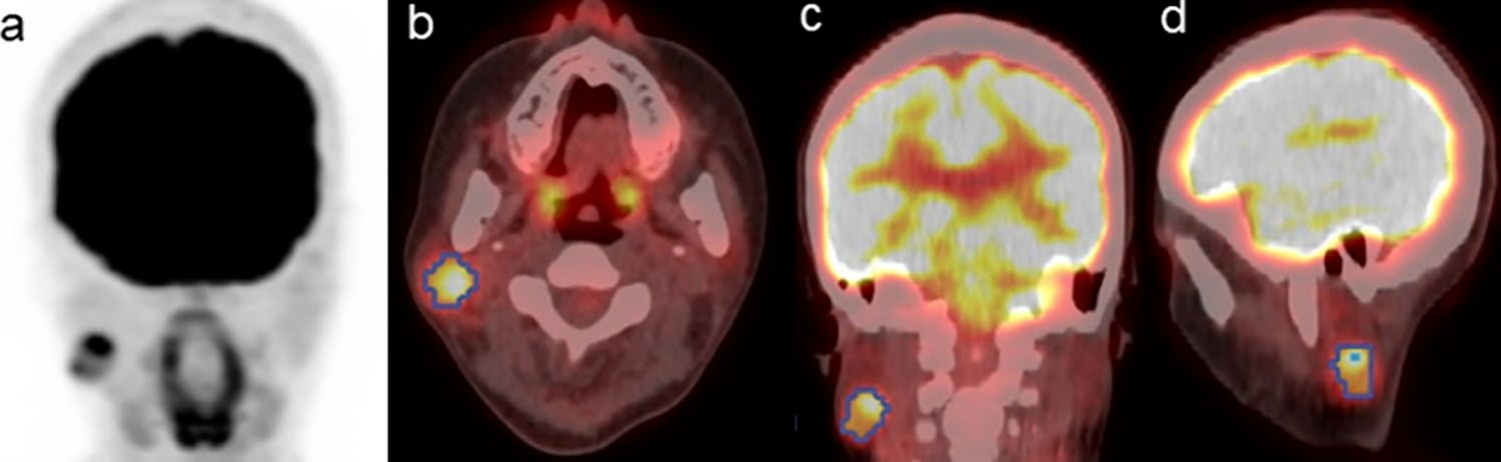
A 33-year-old female patient with a benign right parotid gland tumor (pleomorphic adenoma). Pretreatment 2-deoxy-2-[^18^F]fluoro-d-glucose ([^18^F]-FDG) positron emission tomography/computed tomography scan images [MIP (**a**), transaxial (**b**), coronal (**c**), and sagittal (**d**)] revealed focal [^18^F]-FDG uptake in the primary lesion. The blue line represents the border of the volume of interest. The calculated probability score for predicting malignancy (positive ≥ 0.5) was 0.38 in the DL-based ensemble model. Thus, the DL-based ensemble model can predict benign parotid gland disease in this case

Figures 1 and 2 present the representative [^18^F]-FDG-PET/CT scan images of malignant and benign PGDs, respectively.

## Discussion

This study assessed the effectiveness of pretreatment [^18^F]-FDG-PET-based radiomic features, employing a ML method, to distinguish between benign and malignant PGDs. The DL-based ensemble model demonstrated superior performance, achieving the highest AUC and accuracy among conventional ML and DL-based ensemble models. In this DL-based ensemble model, the MTV and GLSZM features were the primary contributors during the modeling process. Consequently, the DL-based ensemble model utilizing [^18^F]-FDG-PET-based radiomic features proves to be effective in distinguishing benign from malignant PGDs.

Previous researches have evaluated the usefulness of [^18^F]-FDG-PET/CT scan radiomic features in predicting outcomes of salivary gland cancers [12, 13]. Cheng et al. [12] evaluated the role of [^18^F]-FDG-PET/CT scan texture analysis in delivering prognostic information in patients with salivary gland carcinomas. They reported that both high SUVmax and high SUV entropy were significantly associated with worse overall survival. Wu et al. [13] found that GLRLM long-term emphasis on pretreatment [^18^F]-FDG-PET/CT scans was an important independent predictor of progression-free survival in patients treated with interstitial brachytherapy for locally advanced salivary gland carcinoma.

In our study, regarding the differentiation between benign and malignant PGDs, five radiomic features including MTV, GLSZM_ZSE, GLSZM_SZE, GLSZM_GLNU and GLSZM_ZSNU were selected by the RFE feature selection method for constructing the ML model. The patients with malignant PGDs had significantly higher MTV, GLSZM_ZSE, GLSZM_GLNU, and GLSZM_ZSNU than those with benign PGDs. GLSZM_ZSE evaluates the consistency in the arrangement of voxel clusters with the same discretized intensity, with elevated values signifying heterogeneous images [44, 45]. The GLSZM_GLNU analyzes the distribution of zone counts among gray values, with high values indicating that the counts are not uniformly distributed across gray levels [46]. The GLSZM_ZSNU measures how zone counts are distributed over different zone sizes, with higher values reflecting an uneven distribution of counts among the zone sizes [44]. Thus, malignant PGDs had a significantly larger MTV and more heterogeneous [^18^F]-FDG uptake than benign ones.

In our study, among selected five radiomic features, only GLSZM_SZE exhibited no significant differences between benign and malignant PGDs. Although the cause of this difference is unknown, this difference might have been affected by the following different characteristics between the RFE method and univariate statistical tests. The RFE method identifies the most relevant features by repeatedly refining smaller sets, emphasizing their contribution to the model’s predictive power [32]. It selects features based on how well they enhance the overall accuracy of the model, often favoring feature combinations over isolated significance [47]. While univariate statistical tests like the Mann–Whitney U test evaluate features individually, RFE captures complex feature interactions that are beneficial to model predictions but might otherwise go unnoticed [47].

Recently, the applications of ML analyses using [^18^F]-FDG-PET/CT scan images have been reported in the head and neck region [14–24]. ML analyses using pretreatment [^18^F]-FDG-PET-based radiomic features might be valuable approaches for predicting survival in patients with head and neck cancers [16–24]. However, according to our knowledge, no study has yet examined the efficacy of [^18^F]-FDG-PET-based radiomics combined with a ML approach for distinguishing between benign and malignant PGDs.

To avoid the impact of overfitting, our study employed RFE methods to build ML models using the top five selected features, aiming to differentiate between benign and malignant PGDs. In the training cohort, all conventional ML models except LR, and the DL-based ensemble model exhibited good classification performance, showing AUC values greater than 0.80. In the testing cohort, three conventional ML models (neural network, kNN, and SVM) and the DL-based ensemble model had AUC values > 0.80. Although neither the AUC nor accuracy significantly differed among these three conventional ML (neural network, kNN, and SVM) and DL-based ensemble models, the DL-based ensemble model had the best performing classifier as it had the highest AUC (0.976) and diagnostic accuracy (94.7% [18/19]). Moreover, its classification performance was similar in the training and testing cohorts (AUC: 1.000 vs 0.976).

AutoGluon is a framework that attempts to avoid a hyperparametric search to the greatest extent possible by training multiple models concurrently and weighting them using a multilayer stacking strategy to obtain the best predicting model [40, 41]. These processes lead to the optimization of predictive accuracy. In this study, it was used to implement the presented DL-based ensemble model. Further, the DL-based ensemble model using CT scan radiomic features implemented by AutoGluon had the highest performance for predicting response to lenvatinib monotherapy for unresectable hepatocellular carcinoma [48]. A previous study has reported the limitation of [^18^F]-FDG-PET/CT scan in differentiating benign from malignant salivary gland tumors [10]. However, the presented DL-based ensemble model had an excellent predictive performance for discriminating benign from malignant PGDs. Therefore, this ensemble model using [^18^F]-FDG-PET-based radiomic features can overcome the previously reported limitation of [^18^F]-FDG-PET/CT scan for differentiating benign from malignant PGDs.

The radiomics features acquired from CT or magnetic resonance imaging (MRI) were also used for constructing ML models to distinguish between benign and malignant PGDs. Xu et al. [49] examined the usefulness of ML model using CT radiomics features to distinguish between benign and malignant PGDs and reported that the constructed ML model achieved good diagnostic performance with an AUC of 0.835. Wen et al. [50] examined whether the ML model using apparent diffusion coefficient map-based radiomic features was useful for differentiating benign from malignant PGDs, and reported that the constructed ML model achieved good predict performance with an AUC of 0.7637. Moreover, contemporary efforts have employed ML models that incorporate multimodal radiomics features to create predictive models for the head and neck area. Sheikh et al. [51] studied the role of image features in predicting xerostomia caused by radiation therapy in patients with head and neck cancer. Their findings indicated that the ML model, which combined CT and MRI radiomics features of the baseline salivary gland, achieved the highest predictive accuracy for xerostomia. Our analysis exclusively utilized pretreatment [^18^F]-FDG-PET-based radiomic features to construct ML models for differentiating between benign and malignant PGDs. Integrating multimodal imaging radiomics into the ML framework may improve classification accuracy.

This study had several limitations. First, it was a retrospective study conducted with a relatively small cohort from a single institution. Therefore, a multicenter prospective study with a much larger sample size is required to validate and confirm our results. Second, small tumors were excluded from the analysis due to challenges in accurate texture evaluation. As a result, it is difficult to distinguish small benign from malignant parotid lesions when [^18^F]-FDG-PET-based radiomic analyses using ML are not feasible. Indeed, we identified two benign and one malignant small lesions. Further research is required to predict whether these tumors are benign or malignant. Third, two different PET/CT scanners were used, and the lack of imaged-based harmonization might have affected the results of [^18^F]-FDG-PET-based radiomic analyses. Although image-based harmonization is essential, it requires post-processing, which often reduces the spatial resolution in images from the latest devices, resulting in suboptimal image quality for subsequent quantitative and radiomic studies [29]. The utility of post-reconstruction harmonization algorithm with ComBat in the [^18^F]-FDG-PET-based radiomic analysis was demonstrated [29, 52]. Orlhac et al. [29] revealed that ComBat was able to remove batch effect regarding vendor and imaging protocol differences within [^18^F]-FDG-PET/CT images of triple-negative and non-triple-negative breast cancers. Dissaux et al. [52] also revealed the same improvement in the prediction of non-small lung cancer survival within different [^18^F]-FDG-PET/CT images. In our study, the distributions of SUVmax and SUVmean in the liver, representing a normal organ, showed significant differences between the two PET/CT scanners before ComBat harmonization (Supplemental materials, Supplemental Table 3 and Supplemental Figs. 1 and 2). Post-harmonization, these differences were no longer significant, with improved overlap in the parameter distributions (Supplemental Table 3 and Supplemental Figs. 1 and 2). Supplemental Figs. 1 and 2 also illustrate the effectiveness of ComBat harmonization in the liver. Additionally, clinical parameters such as age, sex, and differentiation between benign and malignant cases showed no differences between scanners (Supplemental Table 4), allowing us to apply ComBat harmonization without considering biological covariates. Fourth, only 49 radiomic features extracted using the LIFEx software were included in the ML analyses. However, LIFEx is widely utilized for radiomic analyses in PET/CT scan studies [53, 54]. Lastly, the DL-based ensemble model demonstrated excellent classification performance in both training and testing validations. Hence, a training-test scheme with a larger population is recommended for further model validation.

In conclusion, the DL-based ensemble model using [^18^F]-FDG-PET-based radiomic features can be useful in differentiating benign from malignant PGDs.

## Supplementary Information

Below is the link to the electronic supplementary material.

Supplementary file1 (DOCX 259 KB)
