# Supplementary material for: Applying deep learning-based ensemble model to [18F]-FDG-PET-radiomic features for differentiating benign from malignant parotid gland diseases
Source: Jpn J Radiol. 2024 Sep 10;43(1):91–100. doi: 10.1007/s11604-024-01649-6 (PMC11717794; doi:10.1007/s11604-024-01649-6)
Supplement: Supplementary file 1 — Supplementary file1 (DOCX 259 KB) [file 11604_2024_1649_MOESM1_ESM.docx]

| **Supplemental Table 1.** List of 49 quantitative positron emission tomography-based radiomic features | |
| --- | --- |
| Matrix | Index |
| Shape and first-order features | SUV_max_ |
|  | SUV_mean_ |
|  | SUV_peak_ |
|  | Cardiac metabolic volume |
|  | Cardiac metabolic activity |
|  | Surface area |
|  | Sphericity |
|  | Asphericity |
|  | Compacity |
|  | Kurtosis |
|  | Skewness |
| Gray-level cooccurrence matrix | Inverse difference |
|  | Angular second moment |
|  | Contrast |
|  | Correlation |
|  | Entropy |
|  | Dissimilarity |
| Neighborhood gray-tone difference matrix | Coarseness |
|  | Contrast |
|  | Busyness |
|  | Complexity |
|  | Strength |
| Gray-level run-length matrix | Sort-runs emphasis |
|  | Long-runs emphasis |
|  | Low-gray-level run emphasis |
|  | High-gray-level run emphasis |
|  | Short-run low-gray-level emphasis |
|  | Short-run high-gray-level emphasis |
|  | Long-term low-gray-level emphasis |
|  | Long-term high-gray-level emphasis |
|  | Gray-level nonuniformity |
|  | Run-length nonuniformity |
|  | Run percentage |
| Gray-level size-zone matrix | Small-zone emphasis |
|  | Large-zone emphasis |
|  | Low-gray-level zone emphasis |
|  | High-gray-level zone emphasis |
|  | Small-zone low-gray-level emphasis |
|  | Small-zone high-gray-level emphasis |
|  | Large-zone low-gray-level emphasis |
|  | Large-zone high-gray-level emphasis |
|  | Gray-level nonuniformity |
|  | Normalized gray-level nonuniformity |
|  | Zone-size nonuniformity |
|  | Normalized zone-size nonuniformity |
|  | Zone percentage |
|  | Gray-level variance |
|  | Zone-size variance |
|  | Zone-size entropy |

| **Supplemental Table 2**. *P*-values for comparing each conventional machine learning model and deep learning-based ensemble mode in each diagnostic index for differentiating benign from malignant parotid gland diseases | | | | | | | | | | | | | | | |
| --- | --- | --- | --- | --- | --- | --- | --- | --- | --- | --- | --- | --- | --- | --- | --- |
|  | RF | | | | | NN | | | | kNN | | | LR | | SVM |
|  | NN | kNN | LR | SVM | DLE | kNN | LR | SVM | DLE | LR | SVM | DLE | SVM | DLE | DLE |
| Sensitivity | 1.00 | 1.00 | 1.00 | 0.50 | 1.00 | 1.00 | 1.00 | 0.25 | 1.00 | 1.00 | 0.50 | 1.00 | 1.00 | 0.50 | 0.50 |
| Specificity | 0.13 | 0.50 | 0.50 | 0.13 | 0.13 | 0.50 | 0.50 | 1.00 | 1.00 | 1.00 | 0.50 | 0.50 | 0.50 | 0.50 | 1.00 |
| PPV | 0.15 | 0.55 | 0.63 | 0.22 | 0.13 | 0.37 | 0.33 | 0.89 | 0.95 | 0.92 | 0.47 | 0.32 | 0.42 | 0.29 | 0.85 |
| NPV | 0.52 | 0.69 | 1.00 | 1.00 | 0.16 | 0.80 | 0.44 | 0.40 | 0.35 | 0.64 | 0.61 | 0.27 | 1.00 | 0.14 | 0.79 |
| Accuracy | 0.22 | 0.50 | 1.00 | 0.69 | 0.063 | 0.63 | 0.38 | 0.69 | 1.00 | 1.00 | 1.00 | 0.25 | 1.00 | 0.13 | 0.50 |
| AUC | 0.25 | 0.39 | 1.00 | 0.057 | 0.030 | 0.53 | 0.27 | 0.81 | 0.36 | 0.36 | 0.11 | 0.083 | 0.059 | 0.046 | 0.32 |
| *RF*, random forest; *NN*, neural network; *kNN*, k-nearest neighbors; *LR*, logistic regression; *SVM*, support vector machine; *DLE*, DL-based ensemble model*; PPV*, positive predictive value; *NPV*, negative predictive value; *AUC*, area under the receiving operating characteristic curve | | | | | | | | | | | | | | | |

| **Supplemental Table 3.** Comparison of SUV-related parameters including SUVmax and SUVmean of liver between before and after Combat harmonization | | | | | | | | | | | | | | |
| --- | --- | --- | --- | --- | --- | --- | --- | --- | --- | --- | --- | --- | --- | --- |
| Parameter | Before Combat harmonization | | | | | | | After Combat harmonization | | | | | | |
|  | Discover 600M scanner (n=36) | | | Discover MI scanner (n=26) | | | *p* value | Discover 600M scanner (n=36) | | | Discover MI scanner (n=26) | | | *p* value |
|  | Median | IQR | Range | Median | IQR | Range |  | Median | IQR | Range | Median | IQR | Range |  |
| SUVmax | 3.52 | 3.21-3.92 | 2.69-5.60 | 2.68 | 2.44-2.95 | 1.86-3.74 | <0.001 | 3.11 | 2.92-3.62 | 2.46-5.07 | 3.04 | 2.76-3.44 | 2.36-4.60 | 0.42 |
| SUVmean | 2.53 | 2.37-2.71 | 1.96-3.57 | 2.24 | 2.02-2.49 | 1.59-3.08 | 0.001 | 2.44 | 2.22-2.58 | 1.92-3.41 | 2.39 | 2.19-2.61 | 1.83-3.25 | 0.78 |
| *IQR*, interquartile range | | | | | | | | | | | | | | |

| **Supplemental Table 4.** Patient characteristic comparison between Discovery 600M and Discovery MI scanners | | | | | |
| --- | --- | --- | --- | --- | --- |
|  | Discover 600M scanner | | Discover MI scanner | | *p* value |
| Number | 36 | | 26 | |  |
|  |  | |  | |  |
| Age (years) (mean, range) | 64 | 28-91 | 65 | 31-90 | 0.97 |
|  |  | |  | |  |
| Sex |  | |  | | 0.66 |
| Male | 23 | | 18 | |  |
| Female | 13 | | 8 | |  |
|  |  | |  | |  |
| Differentiation of benignity and malignancy |  | |  | | 0.73 |
| Benign parotid gland disease | 14 | | 9 | |  |
| Malignant parotid gland disease | 22 | | 17 | |  |

**Supplemental figures**

(a)


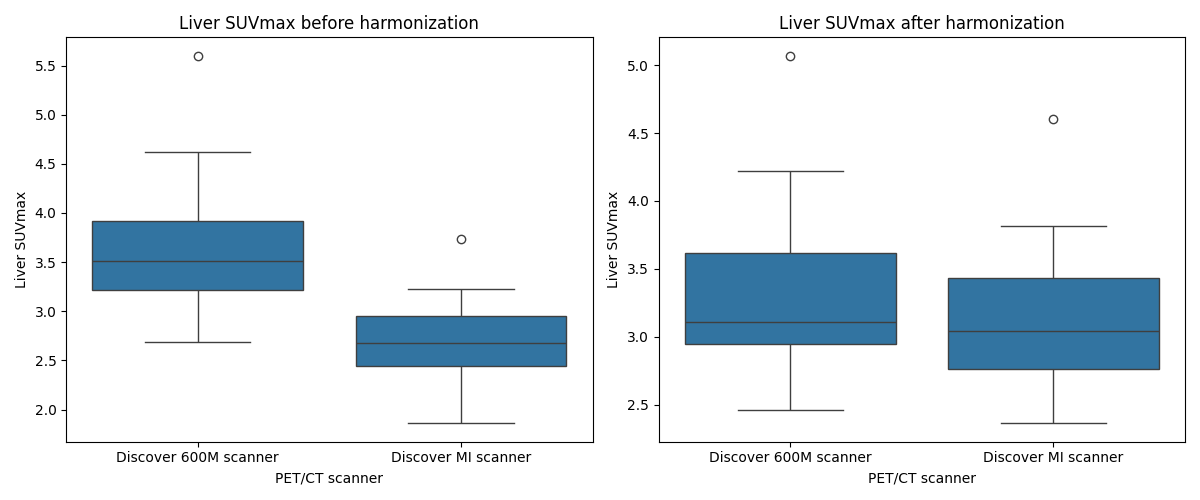


(b)


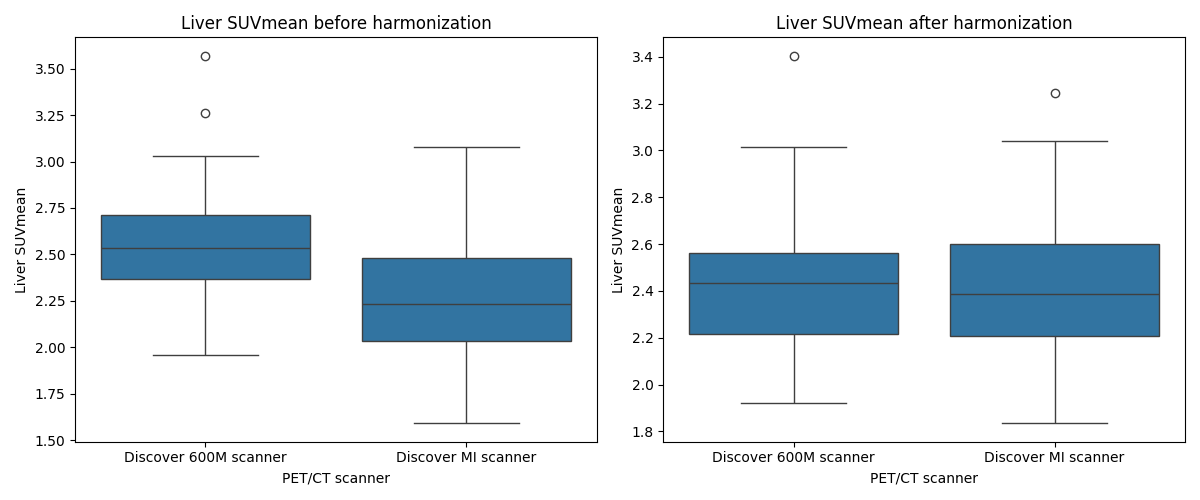


**Supplemental Figure 1.**

Box plots of liver SUVmax (a) and liver SUVmean (b) between 2 PET/CT scanners before and after Combat harmonization.

**(a)**


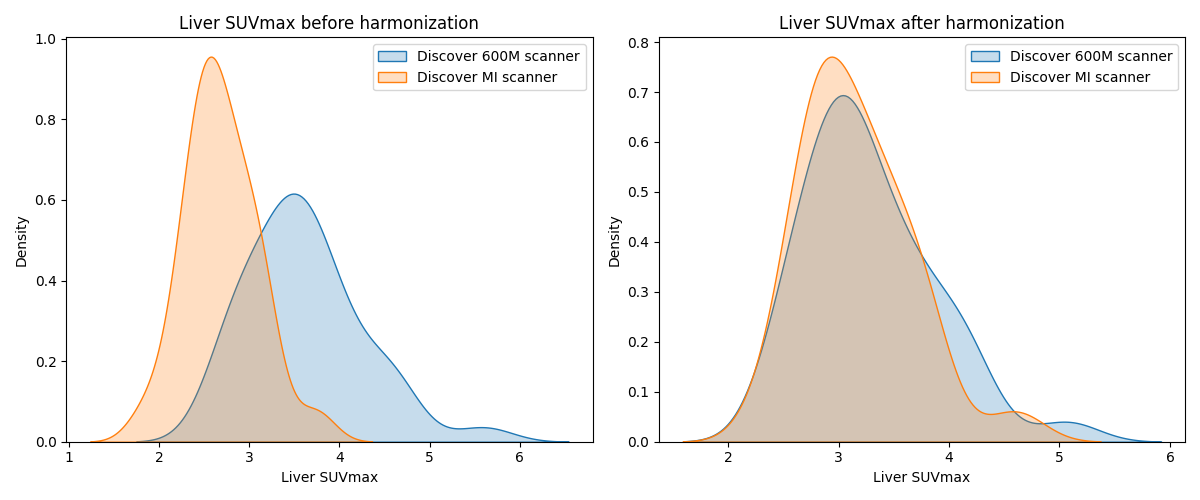


(b)


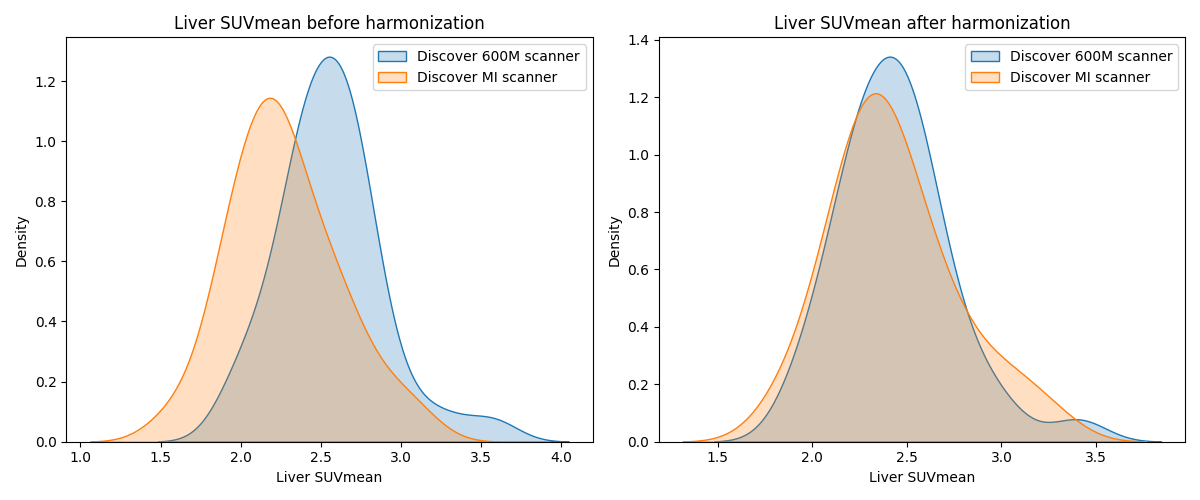


**Supplemental Figure 2.**

Probability density function (%) of liver SUVmax (A) and liver SUVmean (A) before and after Combat harmonization.

**Supplemental material**

**ComBat harmonization methods**

The ComBat model [1] assumes that the value of each feature y measured in VOI j and scanner i can be written as:

$y_{ij}$ =α + $X_{ij}$β + $\gamma_{i}$ + $\delta_{i}$+ $\varepsilon_{\mathrm{ij}}$

α corresponds to the average value of the feature of interest *y*, *X* is the design matrix for the covariates of interest, β is the vector of regression coefficients corresponding to each covariate, $\gamma_{i}$ is the additive effect of scanner *i* affecting the measurement, $\delta_{i}$ is the multiplicative scanner effect, and $\varepsilon_{\mathrm{ij}}$is an error term [2].

ComBat harmonization consists in estimating $\gamma_{i}$ and $\delta_{i}$ using posterior means of empirical Bayes formulation (noted $\gamma_{i}^{*}$and$\delta_{i}^{*}$).^1^ The normalized value of feature *y* for VOI j and scanner *i* is then obtained as:

$y_{ij}^{Combat}=\frac{y_{ij}-\hat{\alpha}-X_{ij}\hat{\beta}-\gamma_{i}^{*}}{\delta_{i}^{*}}$+ $\hat{\alpha}$ + $X_{ij}\hat{\beta}$

where $\hat{\alpha}$and $\hat{\beta}$ are estimators of parameters α and β, respectively.

The harmonization determines a transformation for each feature separately based on the batch (here, scanner) effect observed on feature values. We used ComBat without accounting for any biological covariate (i.e., *X* = 0) because there were no differences between scanners in terms of clinical parameters (Supplemental table 4).

**Setting of VOI for measurement of SUV-related parameters of liver**

The third radiologist located a spheric VOI of about 25cm^3^ in each patient of right posterior lobe of the liver [3].

# REFERENCES

1. Johnson WE, Li C, Rabinovic A. Adjusting batch effects in microarray expression data using empirical Bayes methods. Biostatistics. 2007;8:118–27.
2. Fortin JP, Parker D, Tunc B, Watanabe T, Elliott MA, Ruparel K, et al. Harmonization of multi-site diffusion tensor imaging data. NeuroImage. 2017;161:149–70.
3. Orlhac F, Boughdad S, Philippe C, et al. A postreconstruction harmonization method for multicenter radiomic studies in PET. J Nucl Med. 2018:59:1321-8.
